# Supplementary material for: End Sequence Analysis Toolkit (ESAT) expands the extractable information from single-cell RNA-seq data
Source: Genome Res. 2016 Oct;26(10):1397–410. doi: 10.1101/gr.207902.116 (PMC5052061; doi:10.1101/gr.207902.116)
Supplement: Supplemental Material [file supp_gr.207902.116_Supp_Materials.tar.gz › ESAT-esat_v0.1/doc/html/classumms_1_1core_1_1alignment_1_1_paired_end_alignment_factory.html]

ESAT: umms.core.alignment.PairedEndAlignmentFactory Class Reference


ESAT


- Main Page
- Related Pages
- Packages
- Classes
- Files

- Class List
- Class Index
- Class Hierarchy
- Class Members

All Classes Namespaces Files Functions Variables Enumerator Pages

- umms
- core
- alignment
- PairedEndAlignmentFactory

Public Member Functions |
List of all members

umms.core.alignment.PairedEndAlignmentFactory Class Reference

|  |  |
| --- | --- |
| Public Member Functions | |
| AbstractPairedEndAlignment | getAlignment (boolean fragment, SingleEndAlignment firstMate, SingleEndAlignment secondMate, TranscriptionRead strand) |
|  | |

## Member Function Documentation

|  |  |  |  |
| --- | --- | --- | --- |
| AbstractPairedEndAlignment umms.core.alignment.PairedEndAlignmentFactory.getAlignment | ( | boolean | *fragment*, |
|  |  | SingleEndAlignment | *firstMate*, |
|  |  | SingleEndAlignment | *secondMate*, |
|  |  | TranscriptionRead | *strand* |
|  | ) |  |  |

---

The documentation for this class was generated from the following file:

- java/umms/core/alignment/PairedEndAlignmentFactory.java


---

Generated on Fri Aug 15 2014 15:11:07 for ESAT by  

 1.8.7
